# Five Cases of Azoturia1Read before the Fifteenth Annual Meeting of the Iowa State Veterinary Medical Association, Cedar Rapids, Iowa, January 14 and 15, 1903.

**Published:** 1903-02

**Authors:** A. S. Brodie

**Affiliations:** Cedar Falls, Iowa


### FIVE CASES OF AZOTURIA.[1]

By A. S. Brodie, V.S.,
CEDAR FALLS, IOWA.

Case I.—Bay gelding, down twelve hours before I was called. Treatment: Emptied the bladder with the catheter and took four quarts of blood from the jugular vein. Dissolved 16 drachms of sodium chloride in three gallons of water and gave it subcutaneously. The horse was on his feet twelve hours after. Gave no further treatment and recovery was good.

[1] Read before the Fifteenth Annual Meeting of the Iowa State Veterinary Medical Association, Cedar Rapids, Iowa, January 14 and 15, 1903.

Case II.—Treated the same way, only gave the second injection twenty-four hours. The animal was on her feet the next day.

Cases III. and IV.—Treated the same way and made good recovery. The parts where injected became swollen from the amount of water injected, but all passed away in a few hours.

Case V.—Bay mare, fourteen years old. Had stood three days in the stable, was driven three miles and went down. The gluteal muscles were very much swollen and she was showing abdominal pain. Treated the same as the others. Died three hours after. Postmortem revealed an aneurism in the left iliac artery. Was the aneurism the cause of death? This case had been going lame suddenly at times for two years in the left limb, and would be in great pain and break out in a sweat, but would be all right in twenty-four hours.

### Discussion.

Dr. Heck thinks the most important part of the treatment in azoturia, is good nursing.

Dr. J. W. Scott said that he only seldom catheterizes a male that suffers from azoturia.

Dr. Simpson said that he thinks catharsis is strongly indicated in azoturia. He uses eserine or calomel.

In reply to an inquiry by Dr. Repp, a number of members said that they have a common experience with azoturia in the anterior part of the body without involvement of the hind parts.

Dr. Malcolm thinks diuretics contraindicated in azoturia, but considers it useful to stimulate the bowels and liver.

Dr. McLeod uses potassium bromide internally, to quiet the animal, and makes applications of hot water to the loins.

Dr. C. E. Stewart also reports better results from bromide of potassium than from any other drug.

